# Beyond the Horizon: Unveiling the Frontiers of Rectal Cancer Research and Treatment

**DOI:** 10.7759/cureus.48796

**Published:** 2023-11-14

**Authors:** Reda H Mithany, M Hasaan Shahid, Shenouda Abdallah, Mark Abdelmaseeh, Mina Manasseh, Farid Gerges, Andrew Wanees, Mohamed S Mohamed, Mina W Hakim, Samana Aslam, Nesma Daniel

**Affiliations:** 1 Laparoscopic Colorectal Surgery, Kingston Hospital National Health Service (NHS) Foundation Trust, Kingston Upon Thames, GBR; 2 Surgery, Glangwili General Hospital, Carmarthen, GBR; 3 Surgery, Jaber Al-Ahmad Hospital, Kuwait, KWT; 4 General Surgery, Faculty of Medicine, Assuit University, Assuit, EGY; 5 General Surgery, Torbay and South Devon National Health Service (NHS) Foundation Trust, Torquay, GBR; 6 General and Emergency Surgery, Kingston Hospital National Health Service (NHS) Foundation Trust, Kingston Upon Thames, GBR; 7 General Surgery, Dar El-Salam General Hospital, Cairo, EGY; 8 Orthopaedics, King’s College, London, GBR; 9 Surgery, Minia University Hospital, Minia, EGY; 10 General Surgery, Lahore General Hospital, Lahore, PAK; 11 Medical Laboratory Science, Ain Shams University, Cairo, EGY

**Keywords:** low anterior resection, abdominoperineal resection, rectal polyps, rectal cancer, colorectal cancer

## Abstract

Colorectal cancer, ranking among the most prevalent causes of cancer-related mortality, is an escalating global health concern. The incidence and mortality of colorectal cancer are expected to surge substantially by 2030, posing a significant public health challenge. This article provides a comprehensive overview of rectal cancer, encompassing its epidemiology, anatomical intricacies, pathophysiology, clinical presentation, and diagnosis. The tumor-node-metastasis (TNM) classification system for rectal cancer is detailed, offering crucial insights for staging and treatment decisions. Various treatment modalities are discussed, including surgical approaches, systemic therapies, radiation therapy, and local therapies for metastases. Recent advances in robotic surgery and innovative radiation technologies are explored. Furthermore, prevention strategies are elucidated, focusing on lifestyle modifications and pharmacological interventions that may mitigate the risk of colorectal cancer. The article underscores the importance of understanding rectal cancer for healthcare professionals and patients, enabling informed decision-making and enhanced management of this disease. Prognostic factors are outlined, with survival rates and the prognosis of rectal cancer contingent on several influential elements, highlighting the multifaceted nature of this condition. In conclusion, accurate diagnosis, diverse treatment options, and prevention strategies, including advances like robotic surgery, influence rectal cancer outcomes. A comprehensive overview empowers healthcare professionals and patients to make informed decisions for improved disease management and prognosis.

## Introduction and background

Colorectal cancer, as per Global Cancer Statistics (GLOBOCAN) data, ranks as one of the most prevalent and fatal malignant diseases globally. It stands as the third leading cause of cancer-related mortality and the fourth most commonly diagnosed malignant condition, with its incidence steadily increasing [1]. The global burden of colorectal cancer is anticipated to surge by 60%, resulting in over 2.2 million new cases and 1.1 million deaths by 2030. This escalating trend is marked by significant variations in colorectal cancer incidence and mortality rates, closely correlated with disparities in the Human Development Index [2]. This article aims to provide a comprehensive overview of rectal cancer, including its epidemiology, risk factors, anatomy, diagnosis, treatment options, recent advances, and prevention strategies. It emphasizes the complex nature of the disease and its impact on patient prognosis, with the goal of informing healthcare professionals and patients for more effective disease management.

## Review

Anatomy of the rectum

**Figure 1:**
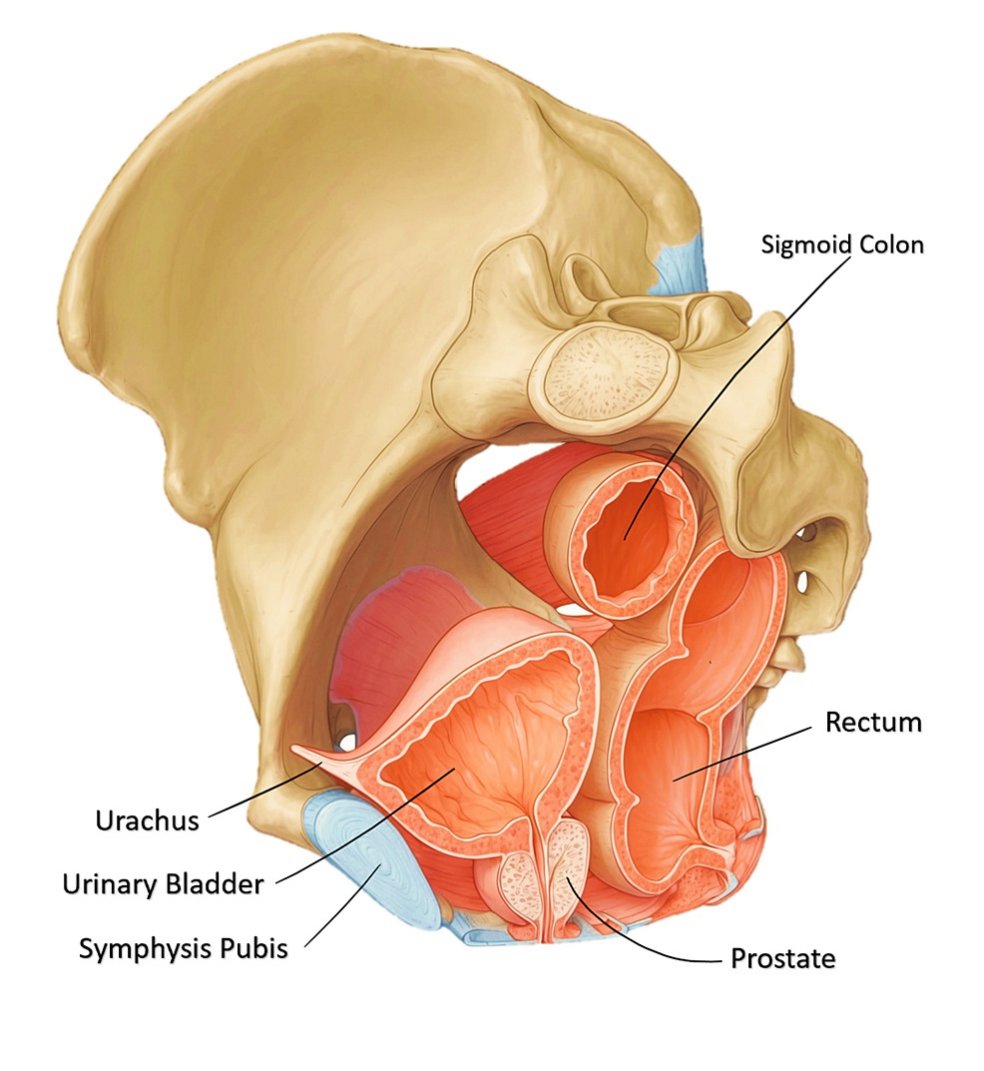
Bladder, prostate, and rectum within pelvic cavity in men (oblique sagittal view) Figure created by Reda Harby Mithany, the article's author.

The rectum is a vital anatomical element within the gastrointestinal tract, commencing at the sacral promontory as a seamless extension of the sigmoid colon. Its external longitudinal muscular layer is a result of the merging of the taenia coli. This gastrointestinal segment exhibits a length spanning from 12 to 15 centimeters, extending from the rectosigmoid junction to the dentate line in the anal canal. The anatomical trajectory of the rectum is marked by two anterior-posterior curvatures, initially following the concavity of the sacrum at the sacral flexure and subsequently bending with an anterior convexity at the anorectal flexure (Figure 1). Additionally, three lateral flexures are shaped by submucosal folds, recognized as the valves of Houston, typically comprising two on the left and one on the right. The terminal portion of the rectum, known as the ampulla, is an expanded segment that rests on the pelvic diaphragm and signifies the transition to the anal canal, where it interfaces with the levator ani muscle [3].

The peritoneal coverage of the rectum exhibits variation; the upper third is covered anteriorly and laterally, the middle third is solely anteriorly covered, while the lower third lacks peritoneal covering. The fascia propria envelops the rectum at this lower level and forms lateral rectal stalks. Posteriorly, the rectum is affixed to the presacral fascia at the S4 level via the Waldeyer fascia. The positioning of the peritoneal reflection fluctuates but is typically situated 6 to 8 centimeters from the anal verge. In males, the reflection extends to the posterior bladder, forming the rectovesical pouch, whereas in females, it extends from the rectum to the posterior cervix, generating the rectouterine pouch, also recognized as the pouch of Douglas. The rectal wall comprises five layers, commencing from the lumen: the mucosa, deep mucosa, submucosa, muscularis propria, and serosa, which is ensconced by the peritoneum. The muscularis propria consists of an inner circular layer that thickens at the anorectal junction, giving rise to the internal anal sphincter, while the outer longitudinal layer continues as the longitudinal aspect of the anal sphincter. Functionally, the rectum predominantly serves as a transient reservoir for feces storage and assumes a pivotal role in the regulation of defecation and the preservation of continence, thus rendering it an indispensable constituent of the gastrointestinal system [3].

From a clinical perspective, colorectal cancers are typically classified into three primary anatomical segments. Proximal or right-sided colon cancers originate in sections proximal to the splenic flexure, including the cecum, ascending colon, and transverse colon. In contrast, distal or left-sided colon tumors develop in regions distal to this site, specifically the descending colon and sigmoid colon. Rectal cancers, on the other hand, are diagnosed when the malignancy emerges within 15 centimeters of the anal sphincter. It is notable that rectal cancers exhibit higher incidence rates of loco-regional relapse and lung metastases, whereas colon cancers tend to display a greater predilection for hepatic dissemination and generally have a relatively more favorable prognosis. The majority of colon cancers fall under the category of adenocarcinomas, which can be further subdivided based on tumor grade into low-grade and high-grade subtypes [4].

Less frequently encountered histological subtypes encompass mucinous adenocarcinomas, adenosquamous carcinomas, signet-cell carcinomas, and medullary carcinomas. It is crucial to underscore that establishing a direct relationship between histological subtype and tumor prognosis is a complex task. Existing evidence indicates that medullary colon cancer is correlated with microsatellite instability (MSI) and appears to be associated with a more positive prognosis, whereas signet-ring cell carcinomas are linked with a less favorable outlook. [4].

The objective of this article is to provide a comprehensive overview of rectal cancer, covering its epidemiology and risk factors, anatomy, pathophysiology, clinical presentation, diagnosis, tumor-node-metastasis (TNM) classification, treatment modalities including surgery, systemic therapy, and local therapies for metastases, and recent advances in the field. It also discusses prevention strategies and the factors influencing the prognosis of rectal cancer patients. This article aims to offer a thorough understanding of rectal cancer for healthcare professionals and patients, facilitating informed decision-making and improved management of the disease.

Epidemiology and risk factors

To effectively address the prevention and management of colorectal cancer, it is paramount to comprehensively understand the associated risk factors. These factors can be categorized into various groups. Gender and race play pivotal roles, with men having a higher risk and often experiencing more unfavorable prognoses than their female counterparts. Notably, non-Hispanic Black individuals exhibit a higher incidence rate compared to other racial groups. Socioeconomic determinants also exert a substantial influence on risk, as individuals with low socioeconomic status face elevated risks due to limited access to healthcare, unhealthy lifestyle habits, and dietary factors. An intricate grasp of these risk factors is indispensable for the prevention, early detection, and effective management of colorectal cancer. Additionally, lifestyle choices play a pivotal role in shaping colorectal cancer risk. Dietary preferences, such as high consumption of red and processed meats and low intake of dietary fiber, fruits, and vegetables, are unequivocally associated with an increased risk. Conversely, adequate intake of calcium, vitamin D, and dairy products may confer a protective effect. The risk is further exacerbated by factors such as overweight and obesity, physical inactivity, and a sedentary lifestyle. Moreover, the consumption of tobacco and alcohol, particularly in excessive quantities, stands as a well-established risk factor [5-9].

Family and personal medical history are substantial contributors to risk. A family history of colorectal cancer, particularly in first-degree relatives, significantly elevates the individual's risk. Hereditary non-polyposis colorectal cancer (HNPCC), also known as Lynch syndrome, and familial adenomatous polyposis (FAP) substantially augment the risk. A personal history of cancer further compounds the risk. Inflammatory bowel diseases, particularly Crohn's disease and ulcerative colitis, are associated with an increased risk, particularly in cases of longer disease duration and greater severity. Colon polyps, particularly adenomatous polyps, possess the potential to progress into malignancy, and approximately 95% of colorectal cancers originate from such polyps. The risk associated with colon polyps escalates with factors like polyp size, degree of dysplasia, and age. It is essential to underscore that alterations in gut microbiota have been implicated in the development of colorectal cancer, engaging diverse mechanisms, including DNA damage and inflammation. Age is a prominent factor, with risk increasing significantly with advancing age, and the majority of cases occurring in individuals over the age of 50. Nevertheless, it is noteworthy that recent trends indicate a rising incidence of colorectal cancer among young adults [5-9].

Pathophysiology

Colorectal cancer (CRC) evolves from the benign proliferation of mucosal epithelial cells, termed polyps, within the large intestine, a process that unfolds over a span of 10 to 20 years before progressing to malignancy. The preponderance of CRC cases take the form of adenocarcinomas, emanating from adenomas that eventually transition into invasive cancer. It is noteworthy that only around 10% of adenomas advance to the invasive stage, although the risk of progression escalates with polyp size. Adenocarcinomas constitute approximately 96% of all CRCs. The staging of the disease plays a pivotal role in determining the prognosis of CRC. In situ, cancers remain confined without invasion into the colon or rectum wall. Local cancers penetrate the wall, regional cancers extend to nearby lymph nodes or adjacent tissues, and distant cancers metastasize to remote organs. The risk of CRC is influenced by a constellation of factors, including dietary choices, lifestyle, genetic predispositions, and mutations in oncogenes and tumor-suppressor genes [10].

Clinical presentation and diagnosis

Rectal cancer is characterized by a spectrum of common clinical manifestations that warrant meticulous consideration. Chief among these presentations is rectal bleeding, often manifesting as hematochezia or melena, involving the excretion of bright red or tarry fecal matter. Disturbances in bowel habits represent a salient diagnostic criterion, encompassing alterations such as diarrhea, constipation, or the sensation of incomplete evacuation. Abdominal discomfort, primarily localized in the lower abdominal region, serves as another hallmark feature. Furthermore, individuals affected by rectal cancer may present concomitant symptoms, including rectal pain, unexplained weight loss, profound fatigue, and anemia characterized by a reduced red blood cell count, leading to a state of debility. Additionally, the presence of narrow stools and tenesmus, which is the persistent urge for defecation following evacuation, merits consideration as potential indicators of rectal neoplasia. It is imperative to underline that while these clinical features are associated with rectal cancer, they may also be attributed to alternative medical pathologies. In view of this, individuals manifesting such clinical indicators or their acquaintances should promptly seek medical evaluation to expedite timely detection and enhance the prospects of favorable therapeutic outcomes [11].

In the diagnostic and treatment journey for rectal cancer, several critical steps are pivotal in providing personalized care. Physicians initiate this process by formulating an individualized treatment plan tailored to each patient's unique circumstances. This plan is meticulously designed, taking into account not only the characteristics of the cancer itself but also the patient's overall health. To facilitate this, a comprehensive health history is compiled, encompassing the patient's medical history and a record of medications administered over their lifetime. This invaluable information aids in making well-informed decisions about the most suitable treatment options. Moreover, a patient's family medical history assumes significant importance, as rectal cancer and related diseases often have a genetic component. Understanding the medical history of blood relatives helps assess the potential risk of inherited cancer syndromes. In some cases, rectal cancer can be associated with inherited genetic mutations, notably Lynch syndrome and FAP. If a patient is suspected of having such a syndrome, they are typically referred to genetic counselors, who can provide further evaluation and facilitate genetic testing [12].

Crucial diagnostic procedures, such as colonoscopy and biopsy, are imperative for confirming the presence of rectal cancer. Colonoscopy, involving the insertion of a colonoscope to examine the colon and rectum, aids in identifying polyps and other abnormalities. During this procedure, biopsy samples are collected, providing insights into the characteristics of the cancer, which are subsequently detailed in a pathology report. Additionally, blood tests, including complete blood counts (CBC) and carcinoembryonic antigen (CEA) tests, assist in detecting signs of disease and monitoring specific markers associated with rectal cancer [12,13].

Imaging tests, such as CT scans and MRIs, are instrumental in visualizing the extent of the cancer, including whether it has spread to other regions. Biomarker testing further refines treatment decisions by identifying targetable genetic or protein changes within the cancer. This information can guide treatment choices and, in some cases, provide eligibility for clinical trials [14].

Another critical consideration for rectal cancer patients, especially given the increasing diagnosis in young adults, is fertility and family planning. Certain cancer treatments, such as radiation therapy, can impact fertility. Consequently, patients of childbearing age are encouraged to engage in discussions about fertility preservation options with their healthcare providers, which may include sperm banking, egg freezing, ovarian tissue banking, or ovarian transposition, preserving the possibility of having children after completing cancer treatment [12].

TNM staging of rectal cancer

Table 1 summarizes the staging of rectal cancer [1,15].

**Table 1:** TNM Classification of Rectal Cancer TNM classification: Tumor-node-metastasis classification The subcategorization of the T3 category is ascertained through an MRI evaluation and is integral to the formulation of treatment recommendations in accordance with European guidelines [15].

Category	Description
T category	
Tx	Primary tumor cannot be assessed
T0	No evidence of a primary tumor
Tis	Carcinoma in situ: intraepithelial or invasion of the lamina propria
T1	Submucosa
T2	Muscularis propria
T3	Subserosa and perirectal tissue
	(T3a): <1 mm
	(T3b): 1–5 mm
	(T3c): 5–15 mm
	(T3d): >15 mm
T4	(T4a): Tumor penetrates to the surface of the visceral peritoneum
	(T4b): Tumor invades or is adherent to other organs or structures
N category	
Nx	Regional lymph nodes cannot be assessed
N0	No regional lymph node metastasis
N1	(N1a): 1 lymph node
	(N1b): 2–3 lymph nodes
	(N1c): Tumor deposit(s) in the subserosa, mesentery, or nonperitonealized perirectal tissues
N2	(N2a): 4–6 lymph nodes
	(N2b): 7 or more regional lymph nodes
M category	
M0	No distant metastasis
M1	(M1a): Metastasis confined to one organ or site (eg, liver, lung, nonregional lymph nodes)
	(M1b): Metastasis in more than one organ and/or site or in the peritoneum

Treatment modalities****

*Surgical Treatment*****

Rectal cancer treatment involves surgical resection, with the choice of surgery tailored to each patient. Transanal surgery is suitable for small rectal tumors that haven't penetrated the muscle layer (T1, N0, M0), preserving bowel function but necessitating vigilant monitoring for potential recurrence. Transabdominal surgery is a comprehensive approach involving abdominal incision, enabling the removal of cancerous tissue, lymph nodes, and meticulous staging. Total mesorectal excision (TME) is commonly employed within transabdominal surgery, focusing on precise mesorectum removal while preserving nerves for post-operative quality of life [12,16-18].

Anastomosis is considered when possible, reconnecting the colon to the anus, offering improved postoperative bowel function. If not feasible, a colostomy becomes necessary, diverting stool through a stoma on the abdomen, either temporarily or permanently. The choice of transabdominal surgery method depends on the cancer location and extent, emphasizing the importance of removing at least 12 lymph nodes for accurate staging [12,19].

There are different types of transabdominal surgery. Low anterior resection (LAR) is suitable for treating tumors located in the mid to upper rectum. In addition to removing the cancerous area of the rectum, part or all of the sigmoid colon, the section of the colon closest to the rectum, may be removed. When feasible, colorectal anastomosis is performed to reconnect the colon to the remaining rectum [12,20]. Abdominoperineal resection (APR) is employed for tumors in the lower rectum that may have extended into the anus or nearby muscle (levator ani). It involves making a second incision between the anus and genitals, known as the perineum and typically results in the removal of the area where the rectum and the colon meet, the rectum itself, and the anus. In some cases, the levator muscles may also be removed, and a permanent colostomy is necessary [12,17].

In the management of rectal cancer, medical professionals often explore minimally invasive surgical options as alternatives to traditional open surgery. Two prominent approaches are laparoscopic and robotic resections. Laparoscopic resections involve small incisions, specialized instruments, and a camera, offering benefits such as reduced pain, shorter hospital stays, and faster recovery, primarily suitable for early-stage cases. In contrast, robotic resections, utilizing systems like the da Vinci Surgical System, provide surgeons with enhanced dexterity, a 3D view, and precise robotic arm movements. They are particularly valuable for complex tumors in the pelvis, combining minimally invasive benefits with the potential to overcome laparoscopy's limitations. Both laparoscopic and robotic resections represent significant advances in rectal cancer surgery, offering quicker recovery and improved outcomes compared to open surgery. The choice between them depends on factors such as cancer characteristics, surgeon expertise, and available resources. Patients and healthcare providers collaborate to determine the most suitable approach for each case, aiming to optimize outcomes while ensuring patient well-being and quality of life [21].

It's important to remember that surgery, while effective in treating rectal cancer, carries potential risks and complications. Short-term and long-term side effects can occur, including anastomotic leaks, urinary issues, scar tissue formation, and hernias. Patients are encouraged to engage in open and detailed discussions with their medical teams to understand the surgical options, potential complications, and the full spectrum of side effects associated with rectal surgery, thereby making informed decisions regarding their treatment [12].

Systemic Therapy

Systemic therapy, a cornerstone of cancer treatment, employs medications that circulate through the bloodstream, targeting cancer cells throughout the body. While effective in eradicating cancer cells, it may also harm healthy cells, causing challenging side effects such as hair loss, skin issues, and mouth sores. The most common administration method is intravenous, where drugs are slowly infused through a vein. This approach encompasses various types of systemic therapy, including chemotherapy, targeted therapy, and immunotherapy. Targeted therapy and immunotherapy, distinct from chemotherapy, are highly efficient against specific cancer types, identified by unique biomarkers. Combinations of systemic therapies, known as regimens, are frequently employed based on factors such as metastasis and tumor biomarker testing results [22].

Chemotherapy: This stands as the primary systemic therapy for rectal cancer. Administered in treatment cycles followed by rest days, this approach permits the body to recover. Cycle duration varies depending on the specific drugs used. Potential side effects, ranging from nausea to hair loss, depend on factors such as drug type, dosage, and treatment duration. Intensive regimens, such as FOLFOX, CAPEOX, FOLFIRI, and FOLFIRINOX, which include oxaliplatin or irinotecan, may have harsher side effects, including nerve damage and abdominal cramping. In cases where these intensive regimens are deemed too severe, alternatives like 5-FU/leucovorin or capecitabine may be recommended, despite the potential for side effects like hand-foot syndrome [12,23].

Targeted therapy: This is a treatment method that utilizes drugs tailored to specific characteristics of cancer cells. These therapies can work by targeting cancer cell receptors, inhibiting overactive proteins, or disrupting the growth of blood vessels within tumors. Often combined with chemotherapy, targeted therapies hold great potential for rectal cancer treatment [24].

Immunotherapy: This therapy enhances the activity of the body's immune system to identify and destroy cancer cells. Immune checkpoint inhibitors are specific drugs used in immunotherapy, including pembrolizumab, nivolumab, dostarlimab-gxly, and ipilimumab, which can effectively treat rectal cancers with specific biomarkers. By blocking immune checkpoint interactions, these drugs allow the immune system to recognize and attack cancer cells [12,25].

Radiation Therapy

Radiation therapy employs high-energy rays to eliminate cancer cells. External beam radiation therapy (EBRT) is the primary method for treating rectal cancer. This approach directs radiation from outside the body to the tumor, minimizing damage to surrounding healthy tissues. Different forms of EBRT, including three-dimensional conformal radiation therapy (3D-CRT), intensity-modulated radiation therapy (IMRT), and stereotactic body radiation therapy (SBRT), are used based on tumor size, location, and other factors. Long-course chemoradiation involves combining external radiation with chemotherapy, enhancing the effectiveness of radiation treatment. Short-course radiation therapy delivers a high dose of radiation in fewer sessions, usually without concurrent chemotherapy. Simulation sessions precede treatment to determine the radiation dose and the number of treatments. The therapy is typically well-tolerated and doesn't cause sensations during administration [12,26].

Other radiation therapy approaches: Radiation can also be applied during surgery (intraoperative radiation therapy) or delivered directly to rectal tumors using a catheter with a radiation source at the tip (endorectal brachytherapy), further expanding the array of radiation therapy options for rectal cancer treatment [12].

Local Therapies for Metastases

In the context of treating metastatic rectal cancer, local therapies emerge as pivotal interventions, directly targeting metastatic tumors that may have spread to various areas, including the liver and lungs. These specialized treatments are typically administered by interventional oncologists or radiologists who harness advanced imaging techniques, including CT, ultrasound, MRI, and PET/CT, to pinpoint and precisely target tumors. Local therapies encompass a range of options, including resection, portal vein embolization, and image-guided ablation [27].

Resection:** **Resection, often referred to as surgery, stands as the primary approach for eliminating metastatic rectal cancer in the liver or lungs. Liver resection entails the surgical removal of the cancerous portion of the liver, whereas lung resection removes affected lung tissue. In cases where metastatic tumors are small, image-guided ablation presents itself as an alternative, offering comparable results with reduced complications and quicker recovery times. When complete removal of metastases isn't feasible, a combined treatment approach, involving both surgery and ablation, may be considered. However, if surgery isn't a suitable option due to associated risk factors or underlying health conditions, alternative local therapies such as ablation may be recommended [28].

Portal vein embolization (PVE): PVE is a minimally invasive procedure aimed at increasing the size of the liver when it is initially deemed too small for surgical intervention following a liver resection. This procedure is performed by an interventional radiologist who inserts a catheter into specific liver veins to block blood flow to the tumor. This, in turn, stimulates the healthy part of the liver to grow larger, enhancing the likelihood of a successful liver resection [29].

Image-guided ablation:** **This is an approach designed to eliminate small liver or lung tumors with minimal collateral damage to surrounding healthy tissues. It can be administered by an interventional radiologist through dedicated needles or by a surgeon during open surgery. Ablation may be used either as a standalone treatment for small tumors that can be completely eradicated or in conjunction with surgery. Common ablative methods include radiofrequency (RFA) and microwave ablation, which target and destroy cancer cells using heat, while cryoablation utilizes cold energy for this purpose. Irreversible electroporation (IRE) and laser ablation are less common alternatives. All ablation techniques deliver precise, targeted energy to the tumor while sparing normal tissues [12,30].

Liver-Directed Therapies

For cases where the cancer predominantly affects the liver, intra-arterial liver-directed therapies come into consideration for liver tumors that are unresponsive to chemotherapy and cannot be surgically removed or ablated. These therapies make use of either chemotherapy beads (chemoembolization) or radioactive spheres (radioembolization) to treat liver tumors. In the case of radioactive spheres, the procedure is known as selective internal radiation therapy (SIRT) or transarterial hepatic radioembolization (TARE). This process, performed by interventional oncologists or radiologists, entails the injection of spheres or beads into an artery leading to the liver tumor(s). These spheres or beads accumulate within the tumor, delivering radiation or chemotherapy directly and inducing cancer cell death. Additionally, they can interrupt the tumor's blood supply [12,31].

Hepatic arterial infusion chemotherapy (HAIC)**: **HAIC represents an alternative form of chemotherapy in which drugs are delivered directly to the liver artery to treat metastases. This method is typically used in conjunction with standard intravenous chemotherapy and necessitates the insertion of a port or pump during liver tumor surgery to facilitate direct drug administration to the liver artery. HAIC is typically performed by medical oncologists with specific expertise in this technique [32].

Stereotactic body radiation therapy (SBRT): SBRT is a specialized radiation treatment modality that delivers high doses of radiation to metastatic sites, typically within five or fewer sessions. This precise treatment approach is utilized to target metastatic rectal cancer in areas such as the liver, lungs, or bones [33].

MRI-LINAC: The MRI-Linac is an innovative radiation therapy device that integrates magnetic resonance imaging (MRI) with a linear accelerator (LINAC). This technology finds application in specific cases, such as during SBRT, and enables the accurate tracking and targeting of tumors in real time, particularly when tumors exhibit movement due to factors like breathing. The MRI-LINAC allows for more effective treatment while minimizing damage to surrounding healthy tissues [12].

Recent Advancements in Rectal Cancer Management

Recent advancements in the management of rectal cancer have witnessed the rapid adoption of robotic surgical systems, particularly in the context of colorectal surgery. Robotic surgery offers technical advantages, including enhanced three-dimensional vision, improved hand-eye coordination, and surgeon-controlled precision, rendering it particularly beneficial for complex rectal cancer resections. Studies, including the recent REAL trial, have demonstrated superior short-term outcomes with robotic surgery, such as reduced positive circumferential margins, fewer conversions to open surgery, and improved postoperative recovery [34].

Prevention strategies

Substantial empirical evidence underscores the potential of several interventions to reduce the incidence of colorectal cancer and adenomatous polyps. These interventions include the use of aspirin, nonsteroidal anti-inflammatory drugs (NSAIDs), cyclooxygenase-2 inhibitors, and hormone therapy. However, the utility of these interventions is somewhat constrained due to the presence of associated adverse effects. Compelling evidence supports the supplementation of calcium, a moderate intake of dairy products, a reduction in red meat consumption, increased levels of physical activity, a decrease in body mass index, and the use of statins as factors significantly correlated with a reduced risk of developing colorectal cancer and adenomatous polyps. In contrast, although increased alcohol consumption and tobacco usage have been linked to an elevated likelihood of colorectal cancer, there is a lack of direct empirical substantiation to confirm that reducing alcohol intake or quitting smoking causally reduces the risk in this context [35].

Survival rates

**Figure 2:**
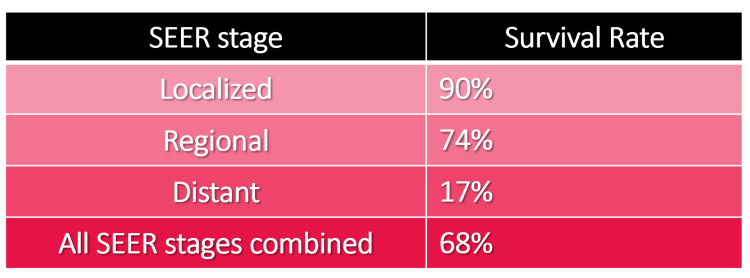
SEER Stage-wise 5 Years Survival Rate SEER program: Surveillance, Epidemiology, and End Results program

The survival rate is defined as the proportion of patients who have been diagnosed with cancer and have survived at a defined period after the diagnosis of the malignant disease. The difference in survival rates varies due to numerous factors, including age, sex, economic status, genetics, histological type, tumor size, stage, comorbidities, and more. In developed countries, the 5-year survival rate for colorectal cancer exceeds 60%. According to the American Cancer Society, the overall 5-year survival rate for rectal cancer patients is 67% (Figure 2) [1].

The prognosis of rectal cancer is influenced by several key factors, including the cancer's stage, tumor size, lymph node involvement, presence of metastasis, histologic grade, molecular and genetic markers, the patient's overall health, response to treatment, completeness of surgery, the expertise of the treatment team, and the emergence of novel therapies such as immunotherapy and targeted treatments. More favorable prognoses are associated with earlier stages, smaller tumors, absence of lymph node involvement, and successful surgical removal of the tumor [36]. A new trend in rectal cancer research and treatment is moving towards personalized medicine, precise therapies, and better use of immunotherapy. Researchers are increasingly focusing on tailoring treatments based on a patient's unique biomarkers and genetics. This approach aims to make treatments more effective while reducing side effects. By bringing together different medical specialists, using advanced surgical methods, and strategies to preserve organs, the goal is to improve patient outcomes and their quality of life. Additionally, providing support, ongoing care, and using health information technology are essential aspects of treating rectal cancer. All of these efforts offer patients a brighter future as the field of rectal cancer research and treatment continues to advance [37].

## Conclusions

In conclusion, rectal cancer is a global health concern with increasing incidence rates and specific risk factors, including gender, race, lifestyle choices, and family history. Accurate diagnosis through colonoscopy, biopsy, and imaging is essential. Treatment options include surgery, systemic therapies (chemotherapy, targeted therapy, immunotherapy), and radiation therapy. Local therapies for metastases are vital, while recent advances in robotic surgery improve outcomes. Prevention involves lifestyle changes and certain medications. The prognosis depends on various factors, with a 5-year survival rate of around 67%. This comprehensive overview aids healthcare professionals and patients in informed decision-making for more effective disease management.
